# The complete mitochondrial genome of a marine mussel, *Modiolus comptus* (Mollusca: Mytilidae), and its phylogenetic implication

**DOI:** 10.1080/23802359.2019.1688728

**Published:** 2019-11-13

**Authors:** Zhen Zhang, Peizhen Ma, Lisha Hu, Yumeng Liu, Haiyan Wang

**Affiliations:** a Department of Marine Organism Taxonomy and Phylogeny, Institute of Oceanology, Chinese Academy of Sciences, Qingdao, P. R. China;; b Center for Ocean Mega-Science, Chinese Academy of Sciences, Qingdao, P. R. China;; c University of Chinese Academy of Sciences, Beijing, P. R. China

**Keywords:** *Modiolus comptus*, mitochondrial genome, phylogeny, Mytilidae

## Abstract

In this study, the complete mitochondrial genome of *Modiolus comptus* was determined and annotated for the first time. The 15,591 bp circular genome has a base composition of 24.3% A, 38.6% T, 12.5% C, and 24.5% G, demonstrating a bias of higher AT content (63.0%) than GC content (27.0%). The mitochondrial genome contains 12 protein-coding genes (PCGs), 20 transfer RNA genes (tRNA), 2 ribosomal RNA genes (*12S rRNA* and *16S rRNA*), and one control region. All genes of *M. comptus* were encoded on the heavy strand, except trnT(ugu) gene. The whole mitochondrial genome of *M. comptus* and 21 mitogenomes of other Mytilidae species were used for phylogenetic analysis. The result indicated the newly sequenced species had the closest relationship with *Modiolus nipponicus* (MK721547) and was clustered within the clade of genus *Modiolus*.

*Modiolus* is a large and diverse genus in Mytilidae (Huber 2010). Although *Modiolus* is not as famous as other economical mussels such as the genus *Mytilus* (Bennion et al. 2019; Naik and Hayes 2019), its economic potential and significance in taxonomy make *Modiolus* a hot spot. Mitogenome has been proven to be an effective tool in understanding the population genetics and relationship of a species (Behera et al. 2016). In this study, the first complete mitochondrion genome DNA sequence of a small, ovate, hairy modiolid species (Qi 2004), *M. comptus*, is determined and described.

The specimen of *M. comptus* was collected on a floating raft outside the wharf of Nanji Island of Zhejiang province, China (27°27′56″N, 121°04′43″E) in June 2012 and deposited in the Marine Biological Museum, Chinese Academy of Sciences (Specimen code: IOCAS_museum_4-02-1462). The complete mitochondrion genome was sequenced via Illumina Hiseq platform and assembled by SPAdes (Wang et al. 2019), and analyzed using MITOS software (Bernt et al. 2013). The complete mitochondrial genome of *M. comptus* was found to be 15,591 bp in length and has been deposited in GenBank (accession No. MN602036). Twelve protein-coding genes (PCGs), 20 transfer RNA genes, 2 ribosomal RNA genes, and 1 control region were identified in the genome. This composition was consistent with most part of genus *Modiolus’* genome, except for *M. modiolus* which contained an additional putative ATP synthase F0 subunit 8 genes (Robicheau et al. 2017). The 12 PCGs found here were cytochrome c oxidase subunit (I, II, and III), NADH dehydrogenase subunit (1, 2, 3, 4, 5, 6, and 4 L), ATP synthase F0 subunit 6, and cytochrome b. The small subunit ribosomal RNA (*12S rRNA*) and large subunit ribosomal RNA (*16S rRNA*) were annotated with sizes of 775 and 1018 bp, respectively. The length of 20 tRNA reported here ranged from 61 to 70 bp. The overall base composition was 24.3% A, 38.6% T, 12.5% C, and 24.5% G, exhibiting a bias of higher AT content (63.0%) than GC content (27.0%). The control region was 670 bp in length and located between trnK(ttt) and 12S rRNA. Among the 34 genes found in this study, 33 genes were encoded on the heavy strand and trnT(ugu) was encoded on the light strand, which was accordant to all previously published species of *Modious*.

Phylogenetic tree involving 22 Mytilidae species with complete mitogenome sequences available in GenBank database was determined based on the datasets of 12 PCGs described previously. Bayesian Inference phylogenies were inferred using MrBayes version 3.2.6 (Ronquist et al. 2012) under partition model (2 parallel runs, 1,000,000 generations), in which the initial 25% of sampled data were discarded as burn-in. As shown in Figure 1, *M. comptus* had the closest relationship with *Modiolus nipponicus* (MK721547) and was clustered within the clade of genus *Modiolus*. Our result also indicated a possibly genetically closer relationship among *Modiolus*, *Bathmodiolus,* and *Limnoperna*, agreed with recent studies of the family Mytilidae (Liu et al. 2018; Lee et al. 2019).

**Figure 1.**
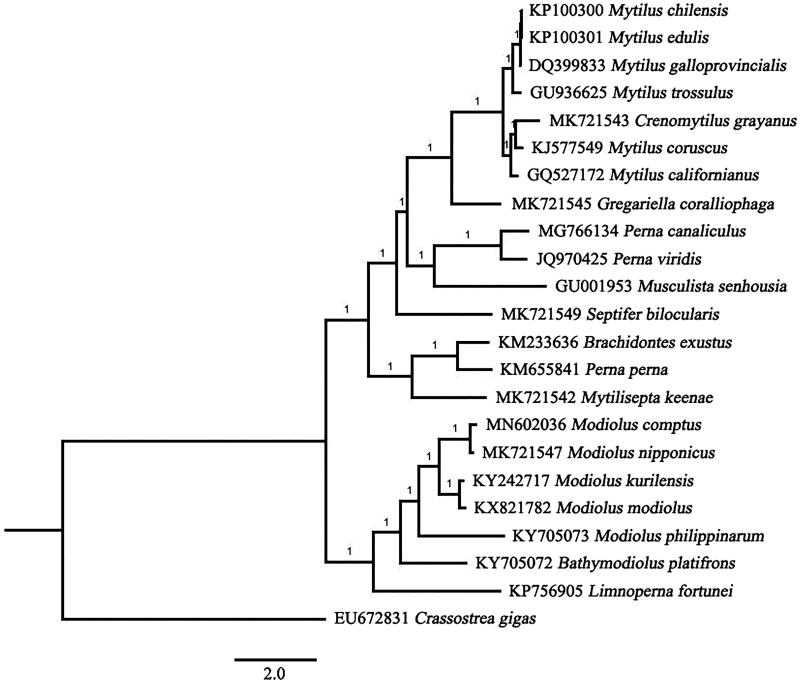
Phylogenetic tree of *M. comptus* and 22 other bivalves using concatenated mitochondrial PCGs.
